# Supplementary material for: Neural mechanism of acute stress regulation by trace aminergic signalling in the lateral habenula in male mice
Source: Nat Commun. 2023 Apr 27;14:2435. doi: 10.1038/s41467-023-38180-7 (PMC10140019; doi:10.1038/s41467-023-38180-7)
Supplement: Supplementary file 1 — Supplementary information [file 41467_2023_38180_MOESM1_ESM.pdf]

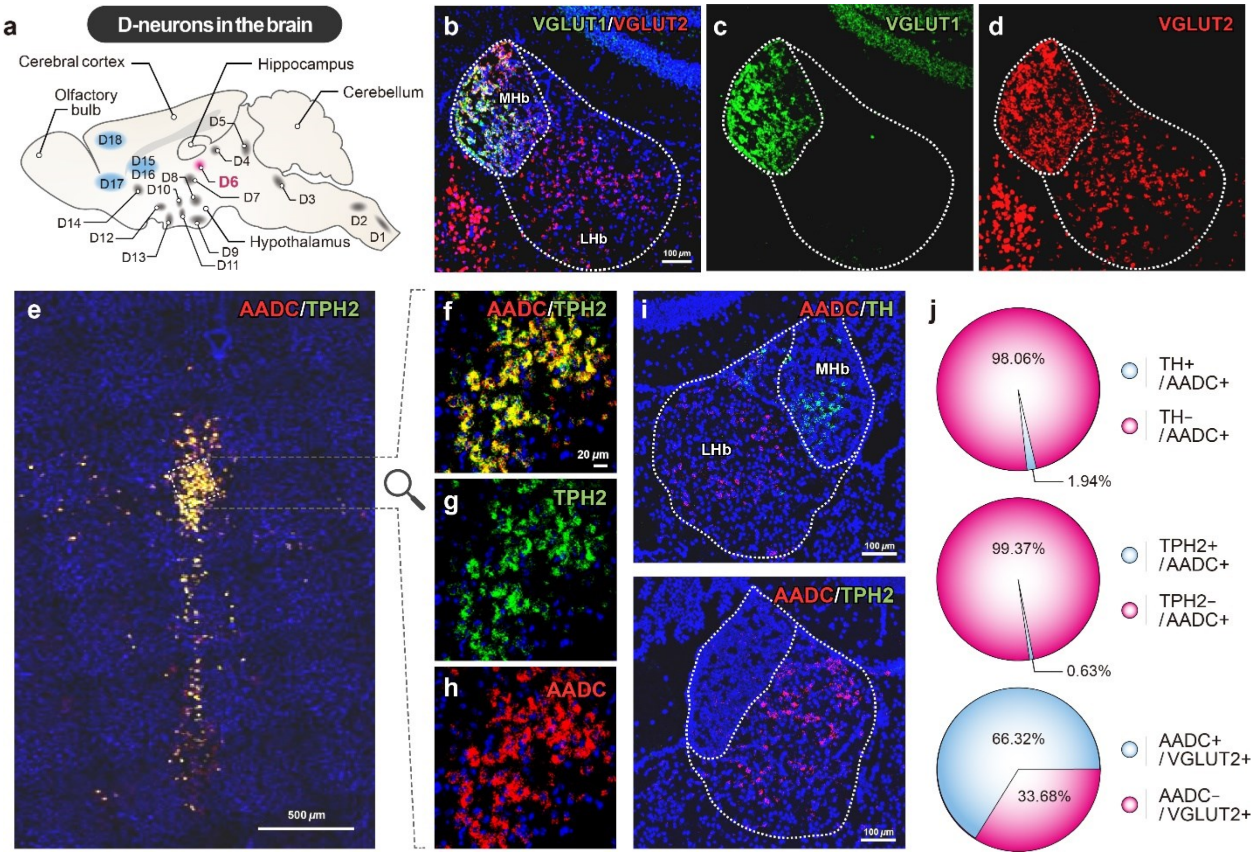

Supplementary Fig. 1: Validation and characterisation of LHb D-neurons. a, Distribution of D-neurons in the brain[64]. D1, spinal cord; D2, nucleus tractus solitarius; D3, parabrachial complex (rostral medulla and pons); D4, midbrain (nuclei associated with the posterior commissure); D5, pretectal nuclei; D6, lateral habenula; D7, paracentral nucleus of the dorsal thalamus; D8, nucleus premammillaris of the hypothalamus; D9, arcuate nucleus; D10, zona incerta; D11, lateral hypothalamic region; D12, dorsomedial hypothalamic nucleus; D13, suprachiasmatic nucleus; D14, bed nucleus of the stria terminalis; D15, striatum; D16, nucleus accumbens; D17, basal forebrain; D18, cerebral cortex. **b**, **c**, Expression of VGLUT1 in the MHb. **b**, **d**, Expression of VGLUT2 in the MHb and the LHb. **e**-**h**, Expression of AADC/TPH2 in the raphe nucleus (**e**), and magnified images of the region of interest (dashed square) (**f**, **g**, **h**). **i**, Expression of AADC, TH and TPH2 in the habenula. **j**, Pie chart of the percentages of TH-expressing AADC-positive cells (n = 4 independent mice), TPH2-expressing AADC-positive cells (n = 4 independent mice) and AADC-expressing VGLUT2-positive cells (n = 3 independent mice) in the LHb by FISH. Source data are provided as a Source Data file.

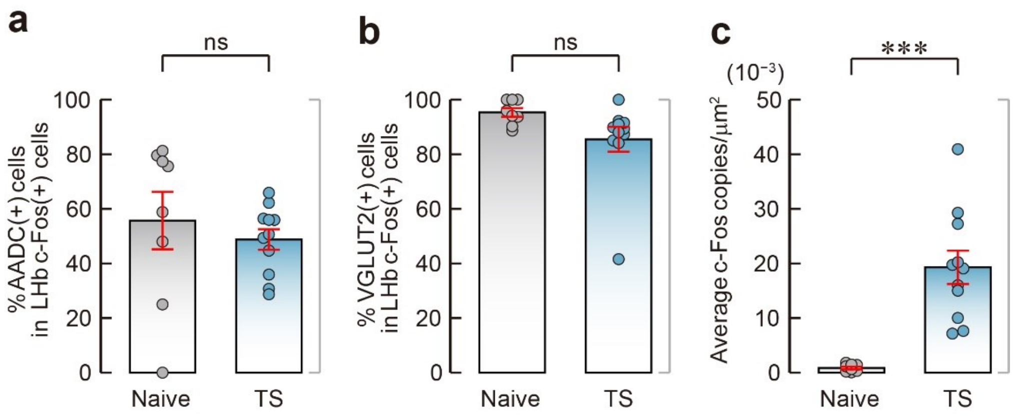

Supplementary Fig. 2: Analysis of activated neurons in the LHb after exposure to tail suspension stress. a, Percentage of AADC-expressing cells among c-Fos-positive cells by FISH cells (n = 8 independent Naïve mice and n = 11 independent TS mice). **b**, Percentage of VGLUT2-expressing c-Fos-positive cells by FISH cells (n = 8 independent Naïve mice and n = 11 independent TS mice). **c**, Mean total number of c-Fos copies per μm^2 in the LHb cells (n = 8 independent Naïve mice and n = 11 independent TS mice). Unless otherwise stated, statistical comparisons were performed using a two-tailed unpaired t test. ***$p < 0.001$. Data are presented as the mean values ± s.e.m. Source data are provided as a Source Data file.

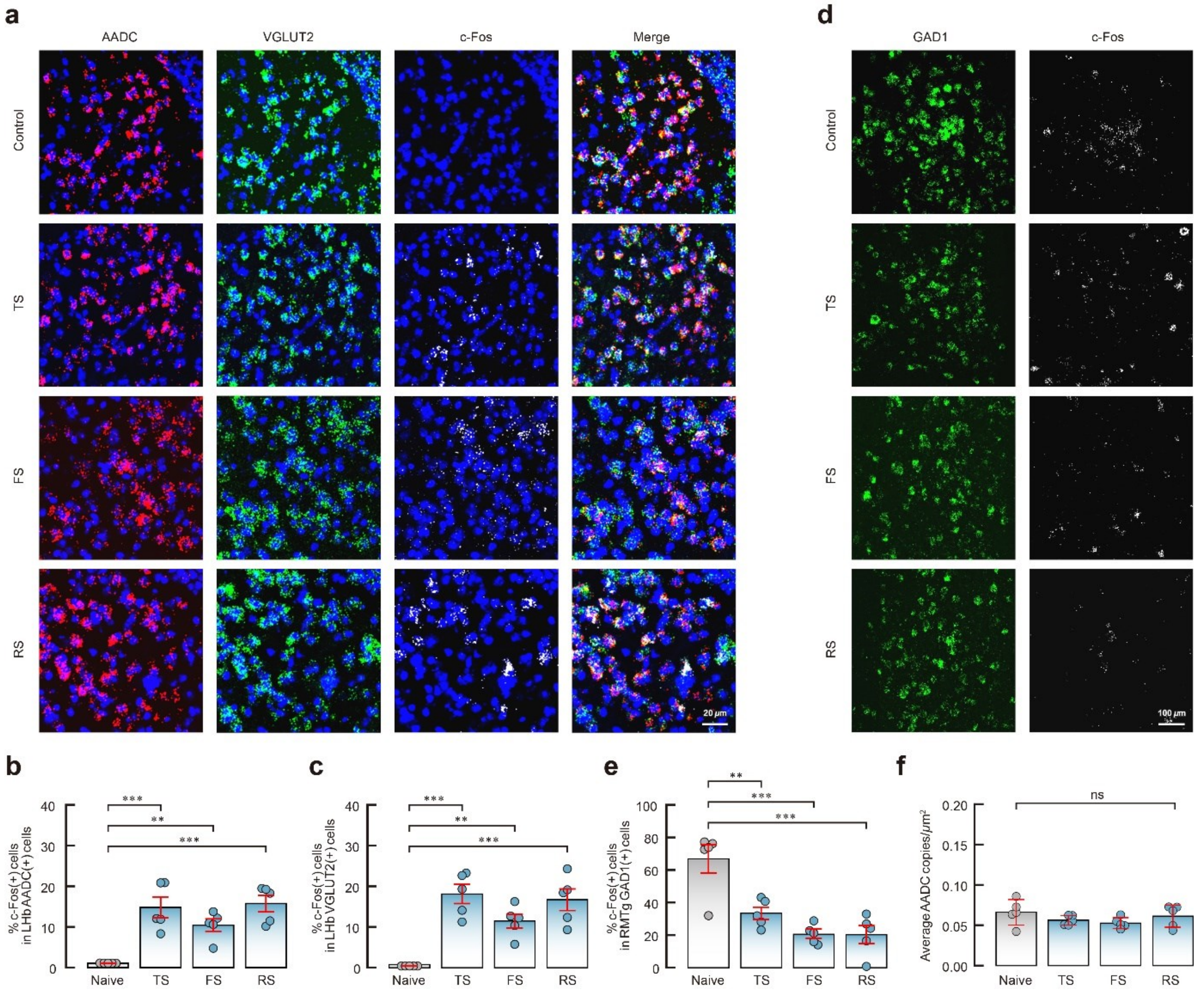

Supplementary Fig. 3: Analysis of the activity of the LHb and the RMTg after various acute stressors. **a**, **d**, Representative images of expression of AADC/VGLUT2/c-Fos in the LHb (**a**) and GAD1/c-Fos in the RMTg (**d**) by FISH. **b**, **c**, **e**, Percentage of c-Fos-expressing AADC-positive neurons (**b**) and c-Fos-expressing VGLUT*2*-positive neurons in the LHb (**c**) and c-Fos-expressing GAD1-positive neurons in the RMTg (**e**) by FISH. **f**, Average total number of AADC copies per μm^2 in the LHb. n = 5 independent Naïve mice, n = 5 independent TS mice, n = 5 independent FS mice and n = 5 independent RS mice. Unless otherwise stated, statistical comparisons were performed using a one-way ANOVA. $^{**}p < 0.01$ and $^{***}p < 0.001$. FS, forced swimming; RS, restraint stress. Data are presented as the mean values ± s.e.m. Source data are provided as a Source Data file.

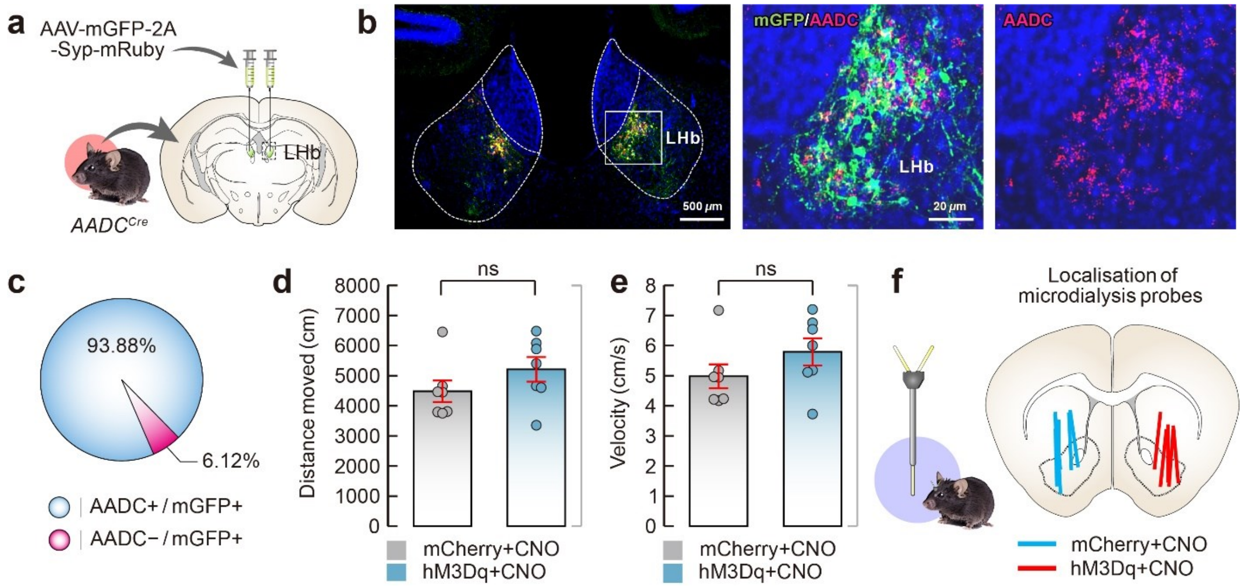

Supplementary Fig. 4: Validation of $AADC^{Cre}$ transgenic mice, effects of chemogenetic stimulation of LHb D-neurons on locomotor activity, and the localisation of microdialysis probes. a, Schematic for the validation of $AADC^{Cre}$ mice using the Cre-dependent virus (a recombinant AAV expressing mGFP/synaptophysin-mRuby). **b**, Representative images of mGFP and AADC mRNA expression in the LHb. Magnified images of the region of interest (white square) are shown to the right of the main image. **c**, Pie chart depicting the proportion of AADC-expressing cells among mGFP-positive cells (n = 3 independent mGFP mice). **d**, **e**, Distance moved (**d**) and velocity (**e**) of mice in the OFT after chemogenetic stimulation of LHb D-neurons (n = 7 independent mCherry mice and n = 7 independent hM3Dq mice), statistical comparisons were performed using a two-tailed unpaired t test. **f**, Schematic of the microdialysis probe placement in the NAc used in the experiment reported in Fig. 2**i**, **j**. Data are presented as the mean values ± s.e.m. Source data are provided as a Source Data file.

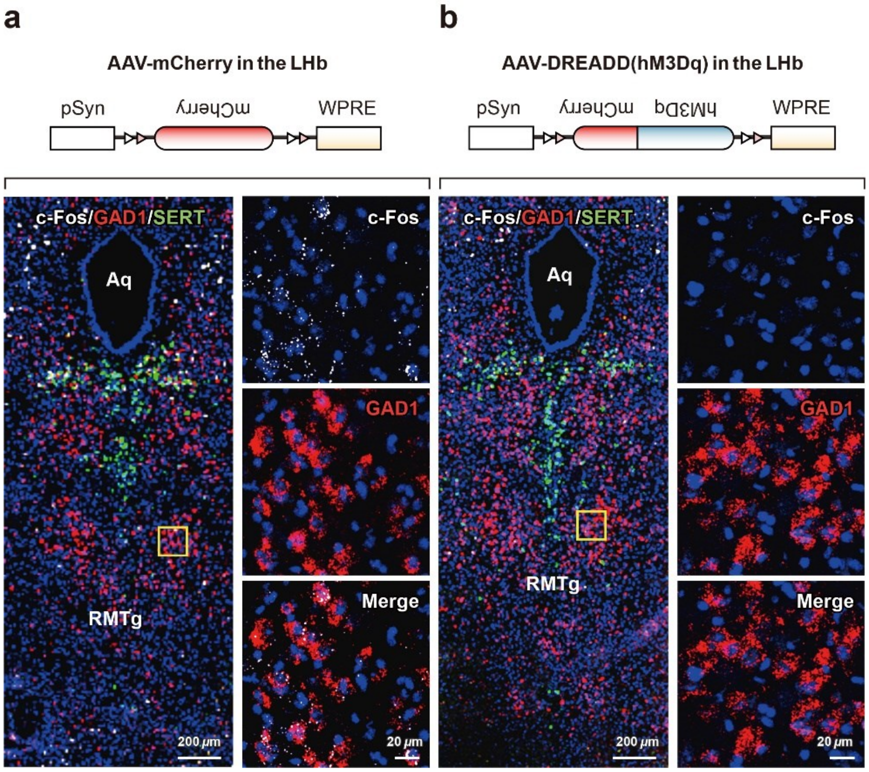

Supplementary Fig. 5: The effect of chemogenetic stimulation of LHb D-neurons on c-Fos expression in RMTg GABAergic neurons. **a**, **b**, mCherry-expressing (**a**) and hM3Dq-expressing (**b**) LHb D-neurons were chemogenetically stimulated, and c-Fos, GAD1 and serotonin transporter (SERT*)* expression were measured in the RMTg by FISH. Magnified images of the regions of interest (yellow squares) are shown to the right of the main images. Aq, aqueduct.

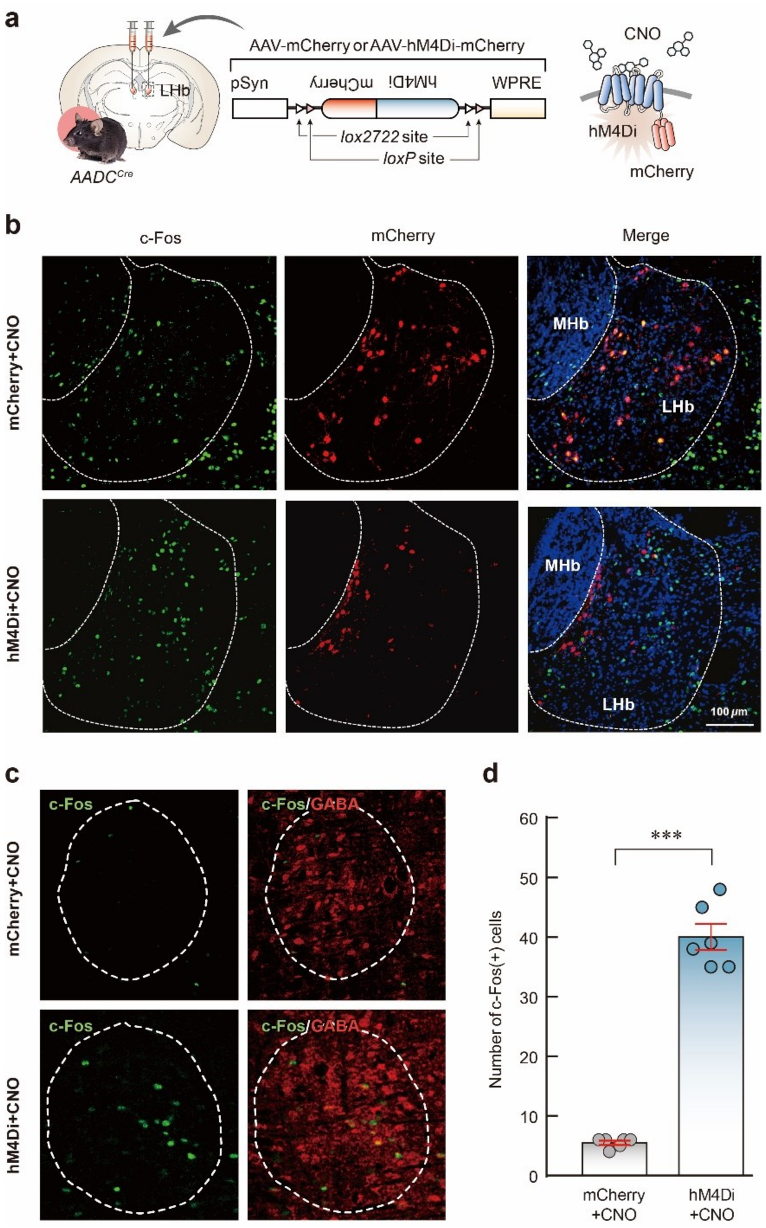

Supplementary Fig. 6: Chemogenetic inhibition of LHb D-neurons during tail suspension stress increases c-Fos expression in the RMTg. a, Schematic of AAV vectors for Cre-dependent hM4Di or mCherry expression **b**, Representative images of viral infection and c-Fos expression in the LHb. **c**, Expression of c-Fos/GABA in the RMTg by immunohistochemistry. **d**, The number of c-Fos-expressing neurons in the RMTg by immunohistochemistry (*n* = 6 independent mCherry mice and *n* = 6 independent hM4Di mice), statistical comparisons were performed using a two-tailed unpaired t test. ***$p < 0.001$. Data are presented as the mean values ± s.e.m. Source data are provided as a Source Data file.

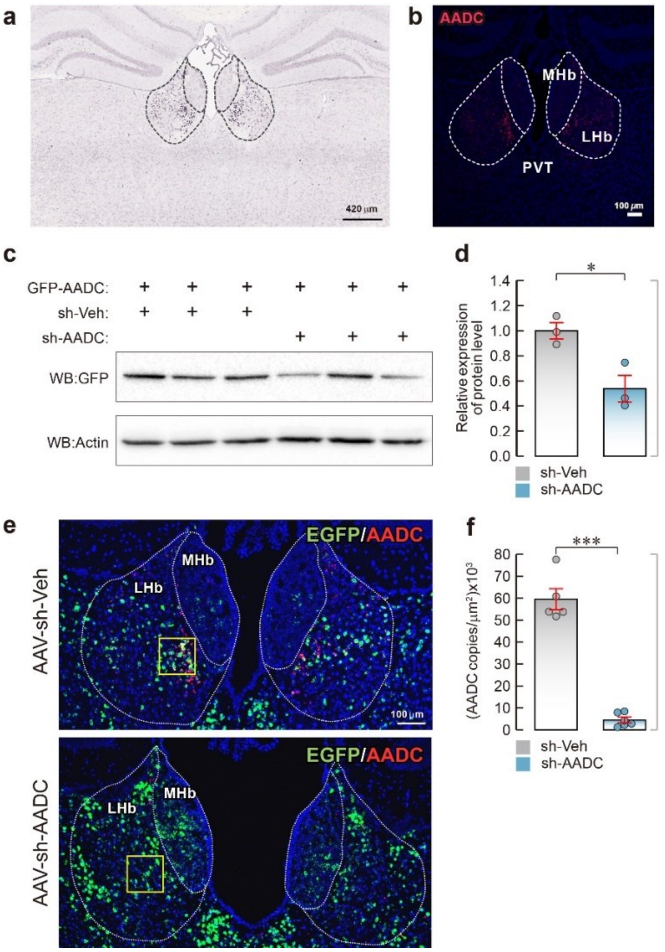

Supplementary Fig. 7: Validation of AADC knockdown in the LHb. a, **b**, Expression of AADC mRNA in the LHb via the Allen Brain Atlas (https://mouse.brain-map.org/) (**a**) and FISH analysis (**b**). PVT, paraventricular nucleus of the thalamus. **c**, **d**, In vitro validation of AADC knockdown in HEK293T cells expressing GFP-AADC (pDEST-CMV-C-EGFP) and shVeh or AAV-shAADC plasmid. After 72 h of expression, the western blot (WB) was imaged (**c**) and the band intensity of exogenous AADC protein levels was normalised to that of actin (n = 3 cell lysates from sh-Veh HEK293T ceclls and n = 3 cell lysates from sh-AADC HEK293T cells) (**d**). **e**, **f**, In vivo validation of AADC knockdown in LHb cells expressing AAV-shVeh or AAV-shAADC for 3 weeks. Representative FISH images of EGFP and AADC in the LHb (**e**). Magnified images of the regions of interest (yellow squares) are shown in Fig. 3**e**. The average number of AADC mRNA copies per μm^2 was multiplied by 10^3 (n = 5 independent sh-Veh mice and n = 6 independent sh-AADC mice) (**f**). Unless otherwise stated, statistical comparisons were performed using a two-tailed unpaired t test. *p < 0.05 and ***p < 0.001. Data are presented as the mean values ± s.e.m. Source data are provided as a Source Data file.

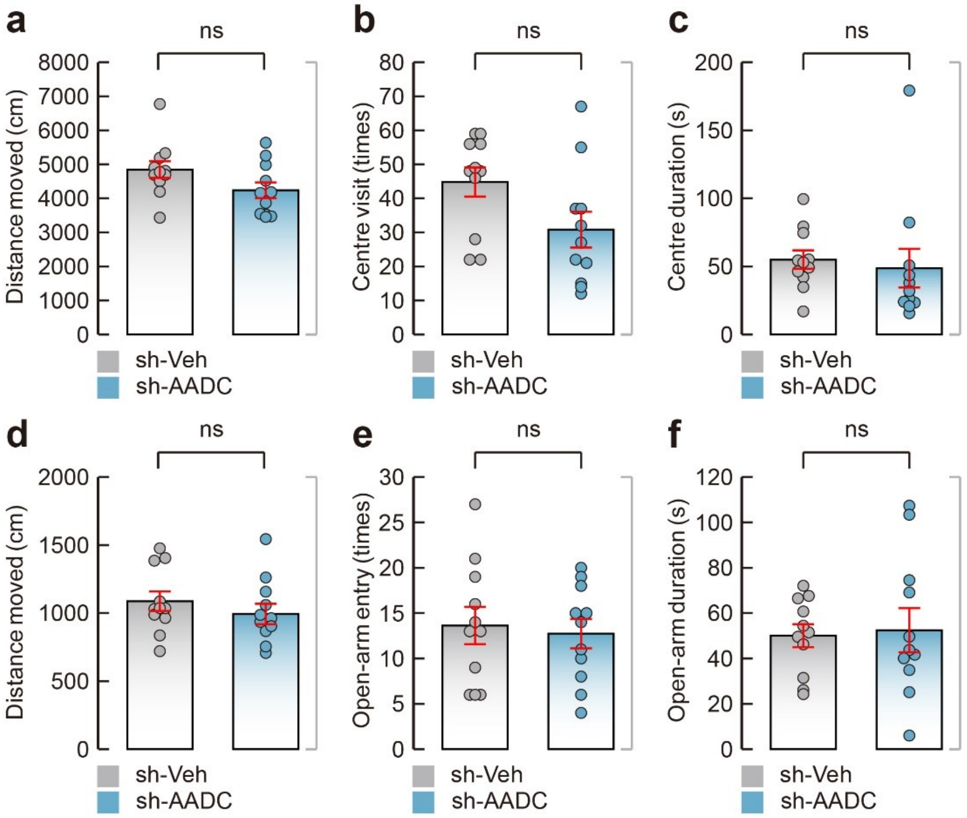

Supplementary Fig. 8: AADC knockdown in the LHb and tests of locomotion and anxiety outcomes. a-c, OFT results: distance moved (**a**), number of centre visits (**b**) and duration spent in the centre (**c**). **d-f**, EZM results: distance moved (**d**), number of entries into open quadrants (**e**) and duration spent in open quadrants (**f**). *n* = 11 independent sh-Veh mice and *n* = 11 independent sh-AADC mice. Statistical comparisons were performed using a two-tailed unpaired t test. Data are presented as the mean values ± s.e.m. Source data are provided as a Source Data file.

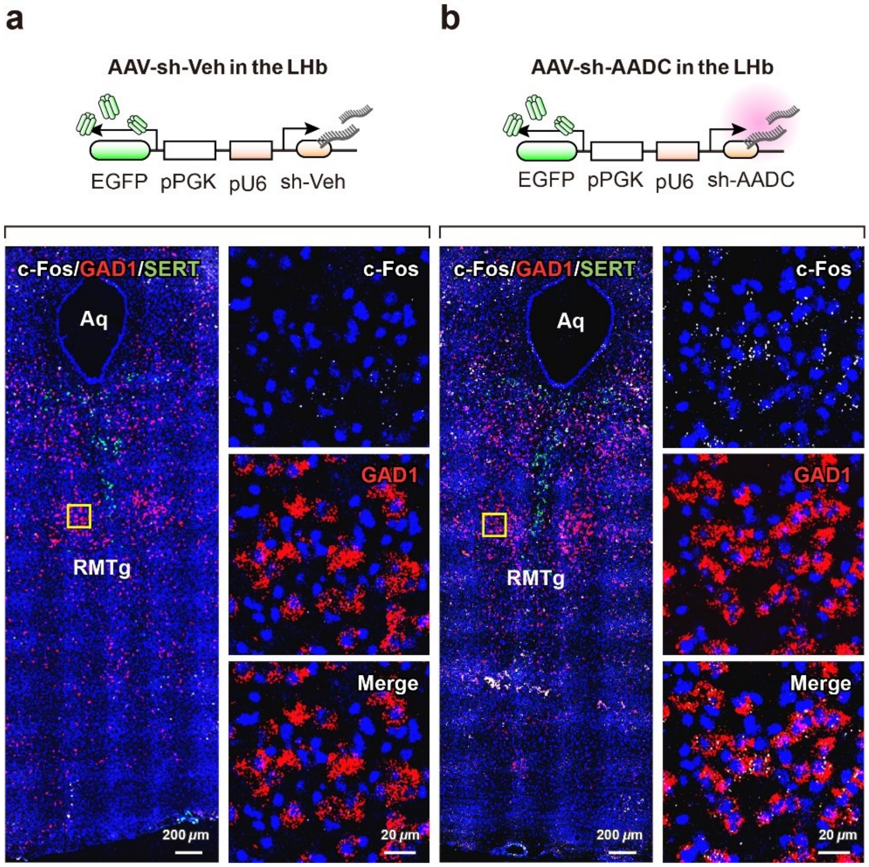

Supplementary Fig. 9: The effect AADC knockdown of LHb D-neurons on c-Fos expression in RMTg GABAergic neurons. **a**, **b**, Vehicle (**a**) and AADC (**b**) were knocked down in the LHb, and c-Fos and GAD1 expression were measured in the RMTg by FISH. Magnified images of the regions of interest (yellow squares) are shown to the right of the main images. Aq, aqueduct.

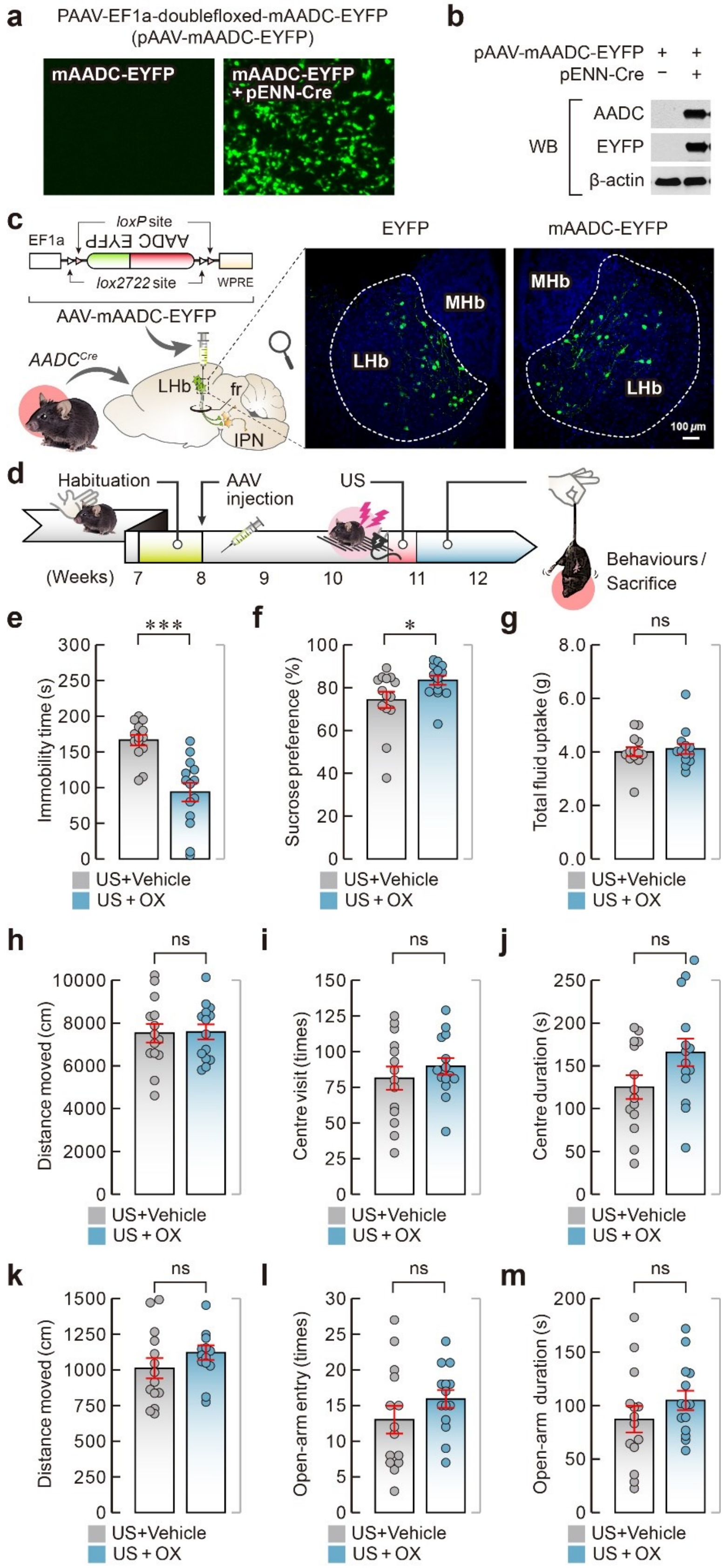
a
PAAV-EF1a-doublefloxed-mAADC-EYFP
(pAAV-mAADC-EYFP)
mAADC-EYFP
mAADC-EYFP + pENN-Cre
b
pAAV-mAADC-EYFP + +
pENN-Cre − +
WB
AADC
EYFP
β-actin
c
loxP site
EF1a
lox2722 site
WPRE
AAV-mAADC-EYFP
AADC^Cre
LHb
fr
IPN
EYFP
mAADC-EYFP
MHb
LHb
100 μm
d
Habituation
AAV injection
US
Behaviours / Sacrifice
(Weeks) 7 8 9 10 11 12
e
Immobility time (s)
***
US+Vehicle
US + OX
f
Sucrose preference (%)
*
g
Total fluid uptake (g)
ns
h
Distance moved (cm)
i
Centre visit (times)
j
Centre duration (s)
k
Distance moved (cm)
l
Open-arm entry (times)
m
Open-arm duration (s)

80

Supplementary Fig. 10: Depressive-like behaviours induced by US with AADC expression in the LHb. a, In vitro validation of AADC overexpression in HEK293T cells expressing mAADC-EYFP and Cre recombinase. **b**, Western blot (WB) analysis of exogenous AADC and EYFP protein levels 72 h after expression. **c**, Schematic of the AAV engineered to overexpress AADC and its location of injection (left). In vivo validation of AADC overexpression demonstrated by the expression of EYFP (right) by FISH. **d**, Experimental paradigm for behavioural assays. **e**-**g**, Effect of AADC overexpression in the LHb on immobility time in the TST (**e**) and on percentage of sucrose preference (**f**) and total fluid intake (**g**) in the SPT. **h**-**j**, OFT results: distance moved (**h**), number of centre visits (**i**) and duration spent in the centre (**j**). **k**-**m**, EZM results: distance moved (**k**), number of entries into the open quadrant (**l**) and duration spent in the open quadrant (**m**). OX, AADC overexpression. n = 14 independent EYFP mice and n = 14 independent mAADC mice. Statistical comparisons were performed using a two-tailed unpaired t test. $*p < 0.05$ and $***p < 0.001$. Data are presented as the mean values ± s.e.m. Source data are provided as a Source Data file.

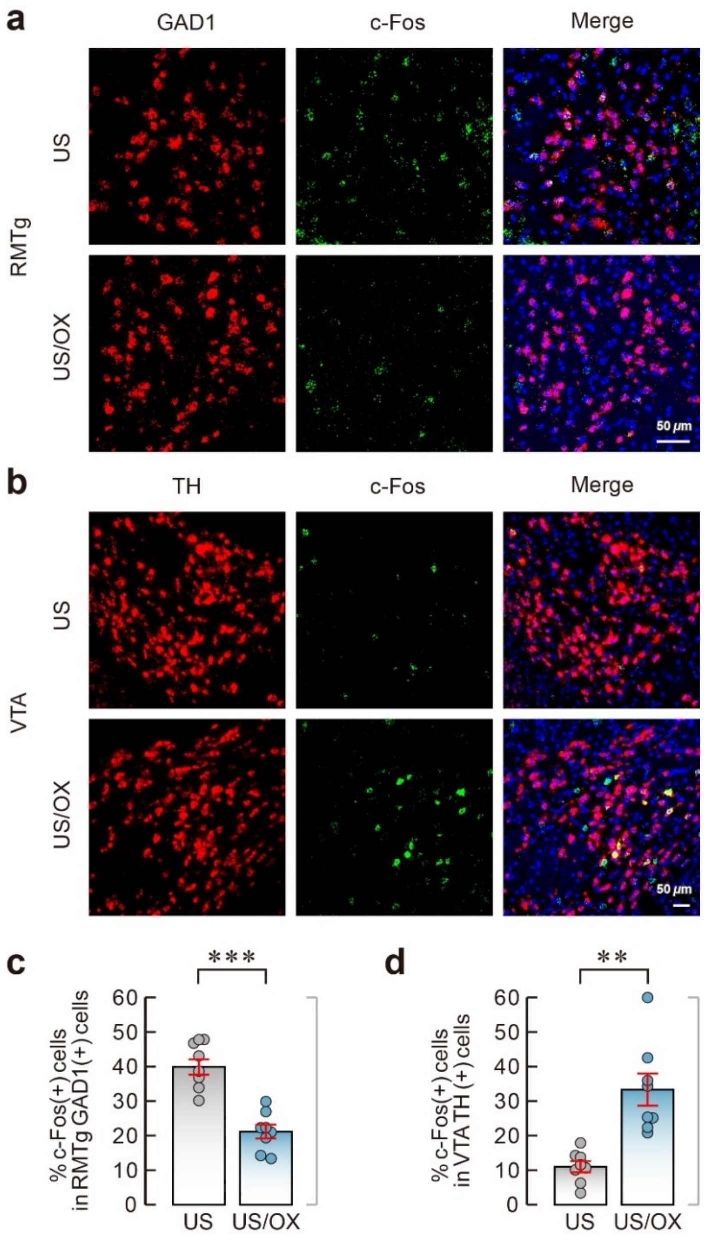

Supplementary Fig. 11: AADC overexpression in the RMTg and VTA after US. a, **b**, Effect of AADC overexpression in the LHb on RMTg GABAergic and VTA dopaminergic neuronal activity. Expression of c-Fos/GAD1 in the RMTg (**a**) and TH/c-Fos in the VTA (**b**). **c**, **d**, Percentage of c-Fos-expressing GAD1-positive neurons in the RMTg (**c**) and c-Fos-expressing TH-positive neurons in the VTA (**d**). $n = 8$ independent EYFP mice and $n = 8$ independent mAADC mice. Statistical comparisons were performed using a two-tailed unpaired t test. $^{**}p < 0.01$ and $^{***}p < 0.001$. Data are presented as the mean values ± s.e.m. Source data are provided as a Source Data file.

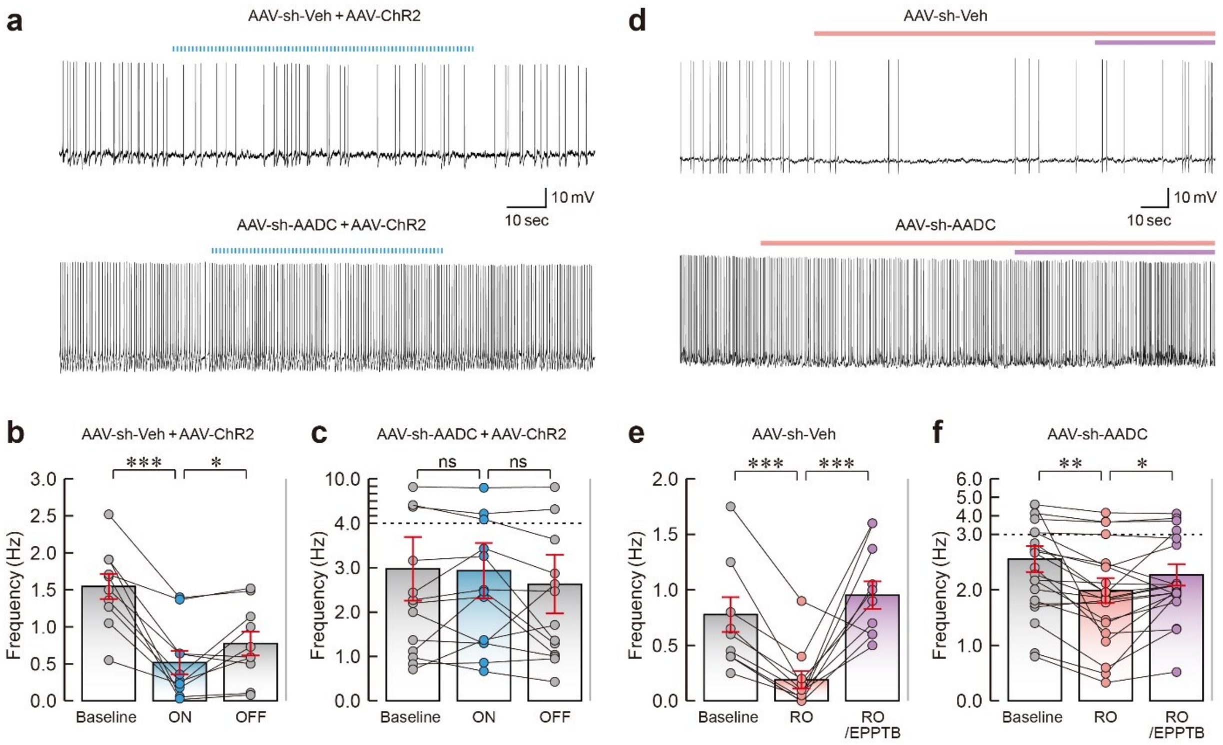

Supplementary Fig. 12: Raw frequency data corresponding to Fig. 5. a, **d**, Full traces by optogenetics for Fig. 5**b** (**a**) and by pharmacological application for Fig. 5**f** (**d**). **b**, **c**, Firing frequencies of neurons in control mice (n = 10 cells from independent sh-Veh/ChR2 mice) (**b**) and AADC-knockdown mice (n = 13 cells from independent sh-AADC/ChR2 mice) (**c**) were measured in the ON and OFF states of blue-light photoactivation. **e**, **f**, Firing frequencies of neurons in control mice (n = 11 cells from independent sh-Veh mice) (**e**) and AADC-knockdown mice (n = 21 cells from independent sh-AADC mice) (**f**) measured after the application of RO5263397 (500 nM) or EPPTB (1 μM). Unless otherwise stated, statistical comparisons were performed using repeated measure one-way ANOVA followed by contrast test. *$p < 0.05$, **$p < 0.01$ and ***$p < 0.001$. Data are presented as the mean values ± s.e.m. Source data are provided as a Source Data file.

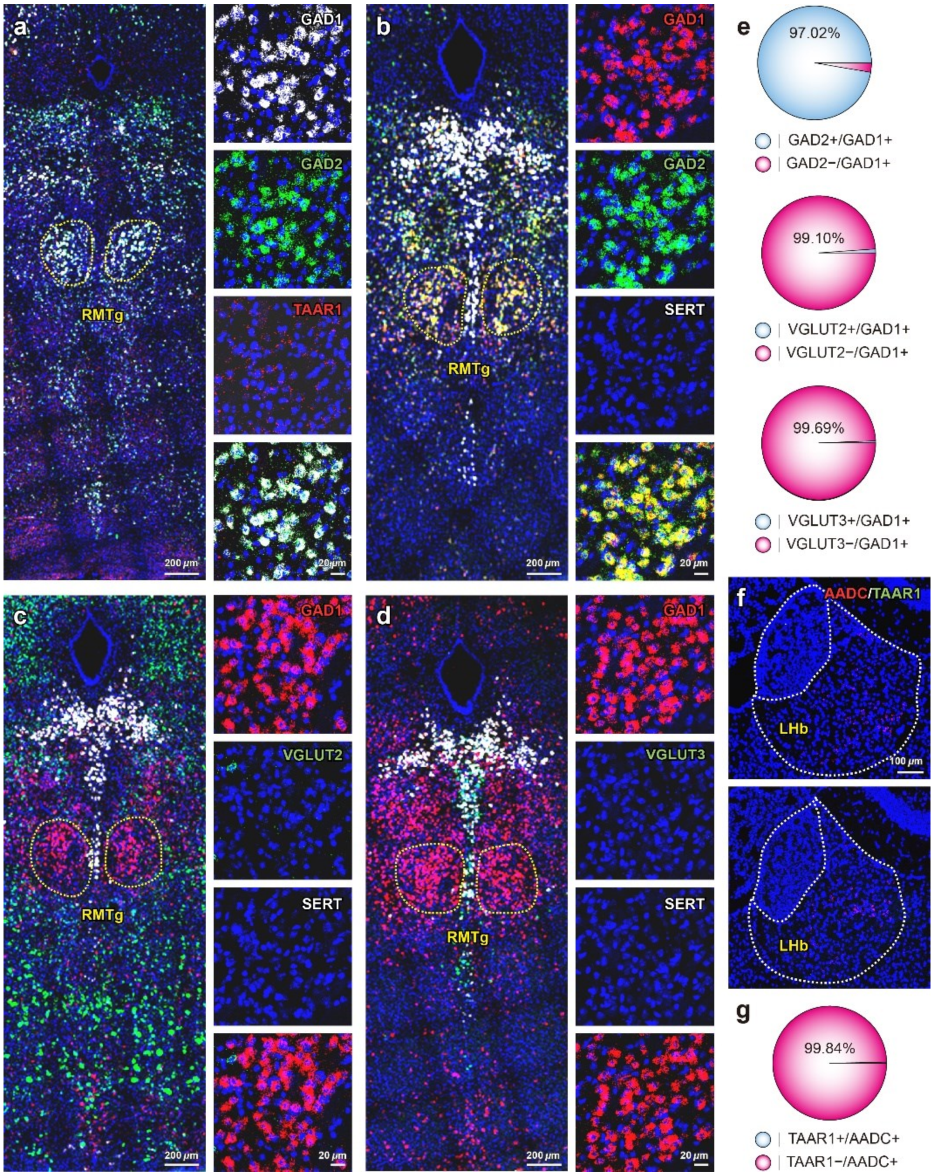

Supplementary Fig. 13: Validation of TAAR1 mRNA expression in RMTg GABAergic neurons and LHb D-neurons. **a**-**d**, Representative FISH images for GAD1/GAD2/TAAR1 (**a**), GAD1/GAD2/SERT (**b**), GAD1/VGLUT2/SERT (**c**) and GAD1/VGLUT3/SERT (**d**) in the RMTg. **e**, Pie charts depicting the percentage of GAD1-expressing neurons co-expressing GAD2 (*n* = 4 independent mice), VGLUT2 (*n* = 3 independent mice) or VGLUT3 (*n* = 3 independent mice). **f**, **g**, Representative images of FISH for AADC/TAAR1 (**f**), and a pie chart depicting the percentage of TAAR1-expressing neurons among AADC-positive neurons (*n* = 3 independent mice) (**g**). Magnified images of the RMTg (dashed yellow circles) are shown to the right of the main images. Source data are provided as a Source Data file.

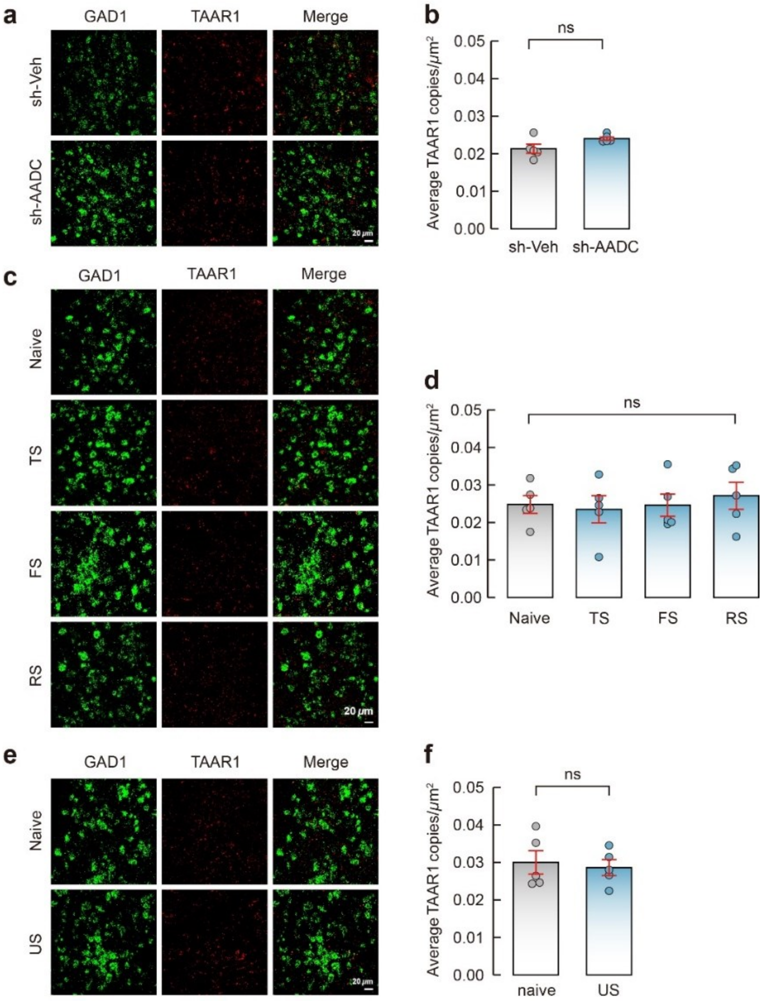

Supplementary Fig. 14: Validation of TAAR1 mRNA expression in RMTg GABAergic neurons after AADC knockdown, US or various acute stressors. a, **b**, Expression of GAD1/TAAR1 in the RMTg (**a**) and the number of TAAR1 mRNA copies per μm^2 in control mice and AADC-knockdown mice (**b**) by FISH (*n* = 5 independent sh-Veh mice and *n* = 5 independent sh-AADC mice). **c**, **d**, Expression of GAD1/TAAR1 in the RMTg (**c**) and the number of TAAR1 mRNA copies per μm^2 in naïve mice and mice treated with the acute stressors (**d**) by FISH (*n* = 5 independent Naïve mice, *n* = 5 independent TS mice, *n* = 5 independent FS mice and *n* = 5 independent RS mice, statistical comparisons were performed using a one-way ANOVA). **e**, **f**, Expression of GAD1/TAAR1 in the RMTg (**e**) and the number of TAAR1 mRNA copies per μm^2 in naïve mice and mice treated with US (**f**) by FISH (*n* = 5 independent Naïve mice and *n* = 5 independent US mice). Unless otherwise stated, statistical comparisons were performed using a two-tailed unpaired t test. Data are presented as the mean values ± s.e.m. Source data are provided as a Source Data file.

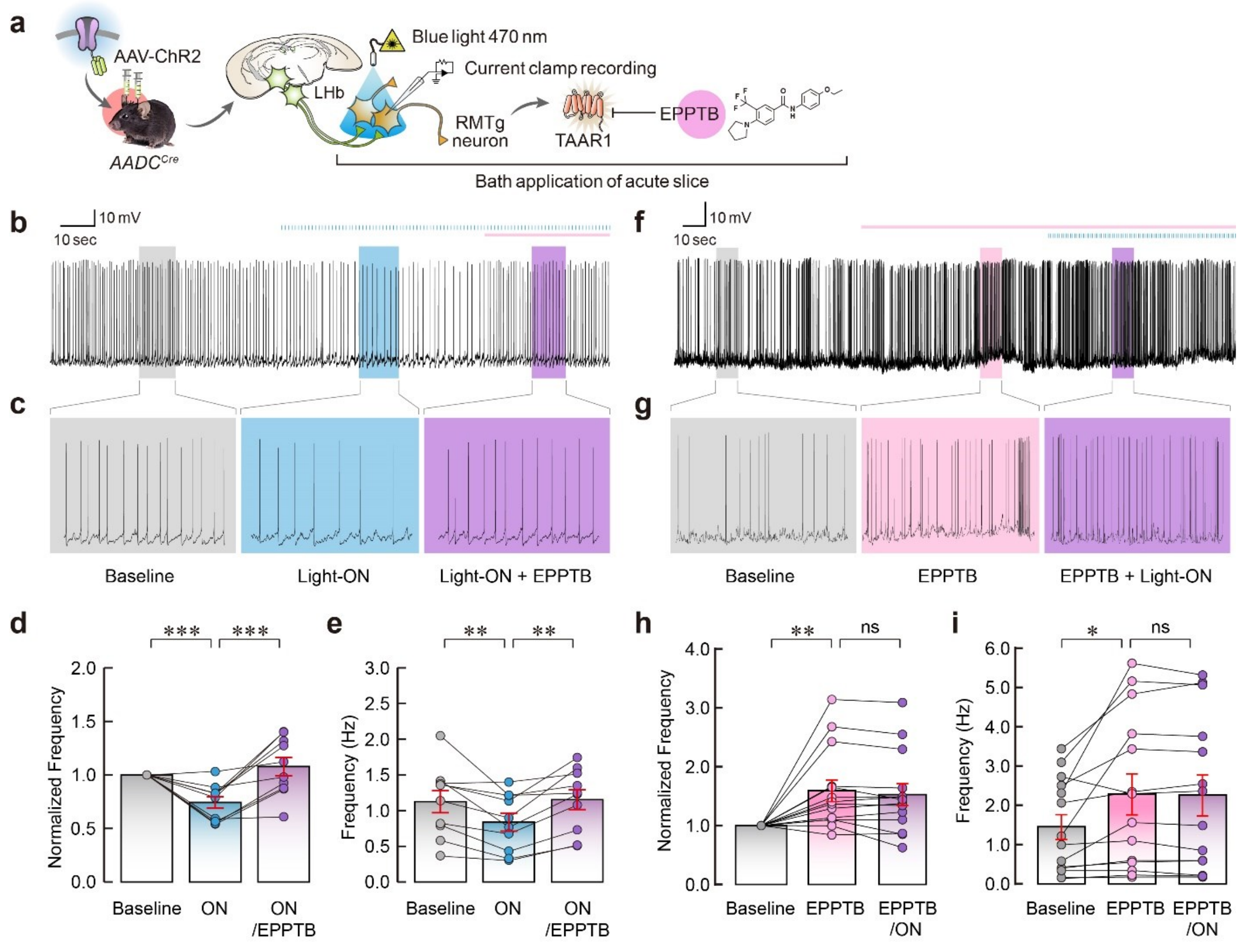

Supplementary Fig. 15: RMTg firing frequencies by optogenetic stimulation of LHb D-neuron terminals before or after EPPTB treatment into the RMTg. a, Schematic of AAV injection and RMTg GABAergic neuronal firing rates in response to the post- or pre-application of EPPTB during optogenetic stimulation of LHb D-neuron terminals. **b**, **f**, Full traces acquired by whole-cell recording from RMTg GABAergic neurons. **c**, **g**, Representative traces measured in response to the application of optogenetic stimulation or EPPTB (1 μM). **d**, **e**, **h**, **i**, The firing frequency was normalised to the baseline value (**d, h**) and measured after the application of optogenetic stimulation or EPPTB (1 μM) (**e, i**). n = 10 cells from independent ON/EPPTB mice and n = 14 cells from independent EPPTB/ON mice. Statistical comparisons were performed using repeated measure one-way ANOVA followed by contrast test. *$p < 0.05$, **$p < 0.01$ and ***$p < 0.001$. Data are presented as the mean values ± s.e.m. Source data are provided as a Source Data file.

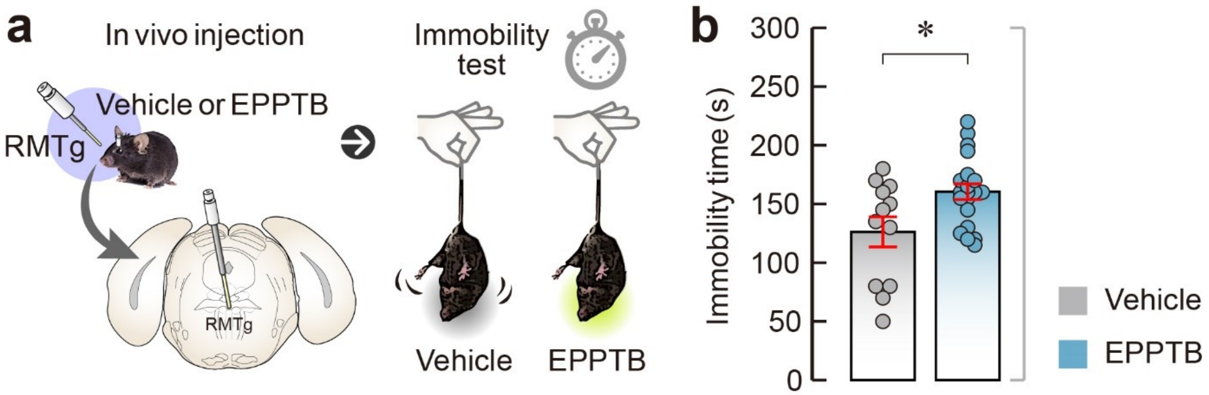

Supplementary Fig. 16: Treatment of EPPTB into the RMTg and quantification of despair-like behaviour. a, **b**, Schematic of cannula implantation over the RMTg (**a**) and TST immobility time (**b**). n = 12 independent Vehicle mice and n = 20 independent EPPTB mice. Statistical comparisons were performed using a two-tailed unpaired t test. *$p < 0.05$. Data are presented as the mean values ± s.e.m. Source data are provided as a Source Data file.

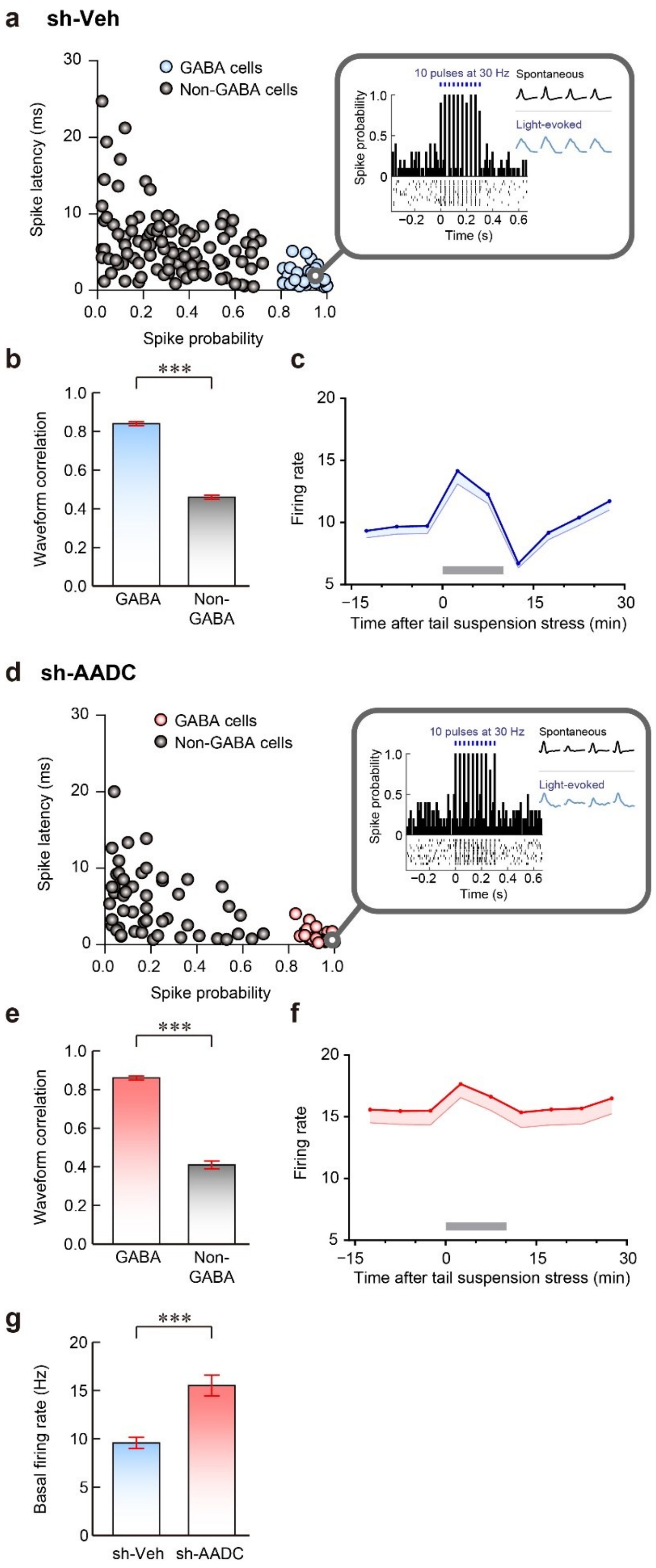

a sh-Veh
GABA cells
Non-GABA cells
Spike latency (ms)
Spike probability
10 pulses at 30 Hz
Spontaneous
Light-evoked
Time (s)
b
Waveform correlation
***
GABA
Non-GABA
c
Firing rate
Time after tail suspension stress (min)
d sh-AADC
GABA cells
Non-GABA cells
Spike latency (ms)
Spike probability
10 pulses at 30 Hz
Spontaneous
Light-evoked
Time (s)
e
Waveform correlation
***
GABA
Non-GABA
f
Firing rate
Time after tail suspension stress (min)
g
Basal firing rate (Hz)
***
sh-Veh
sh-AADC

Supplementary Fig. 17: In vivo recording of RMTg GABAergic neurons before and after exposure to tail suspension stress. a, d, Characteristics of light-evoked responses in control (**a**) and AADC-knockdown (**d**) mice. Peristimulus time histograms (10-ms bins) constructed during the presentation of 10 trains of 10 blue-light pulses (each pulse 5-ms width at 30 Hz; inter-train interval of 60 sec). Spike probability and latency in response to 100 pulses calculated from all RMTg neurons recorded in each group. The boxed histograms show firing patterns of two representative GABAergic neurons. **b, e**, Correlations between spontaneous and light-evoked waveforms of RMTg neurons in control (n = 34 independent GABA cells and n = 103 independent non-GABA cells) (**b**) and AADC-knockdown (n = 29 independent GABA cells and n = 53 independent non-GABA cells) (**e**) mice. **c, f**, Average firing rates of GABAergic neurons (5-min bins) before, during and after tail suspension in control (**c**) and AADC-knockdown (**f**) mice. **g**, Comparison of basal firing rates measured during a 15-min period prior to tail suspension between control and AADC-knockdown mice (n = 34 GABA cells from independent sh-Veh mice and n = 29 GABA cells from independent sh-AADC mice). Unless otherwise stated, statistical comparisons were performed using a two-tailed unpaired t test. ***$p < 0.001$. Data are presented as the mean values ± s.e.m. Source data are provided as a Source Data file.

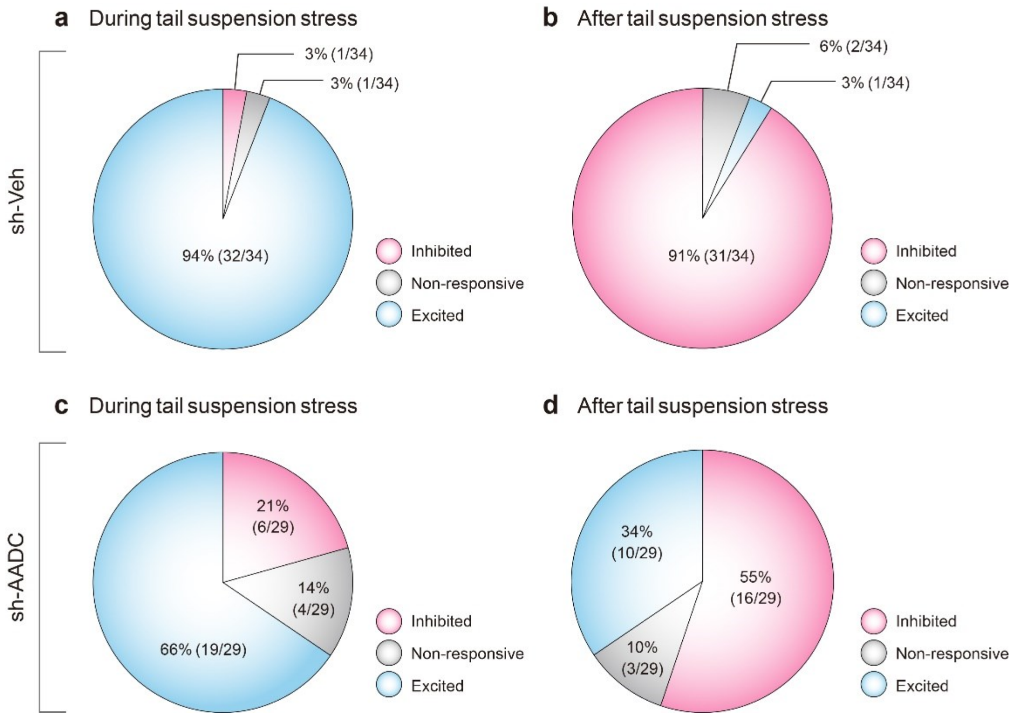

Supplementary Fig. 18: Pie charts showing the percentage of RMTg GABAergic neurons during and after tail suspension stress. a-d, Proportions of GABAergic neurons showing excited, inhibited and no responses during tail suspension (first 5 min; **a** and **c**) and after the stress (first 5 min; **b** and **d**) in control and AADC-knockdown mice, respectively.

170 **Supplementary Table 1. RNAscope FISH probes used in this work.**

Gene name	Accession #	Target region	Cat. #	Manufacturer
AADC	NM_001190448.1	297 – 1726	464871	ACD (Advanced cell Diagnostics)
		297 – 1726	464871-C3	
		835 – 1779	318681-C2	
		835 – 1779	318681-C3	
VGLUT1	NM_182993.2	464 – 1415	416631-C3	
VGLUT2	NM_080853.3	1986 – 2998	319171-C2	
VGLUT3	NM_182959.3	781-1695	431261	
TPH2	NM_173391.3	1640 – 2622	318691-C2	
TH	NM_009377.1	483 – 1603	317621-C2	
TAAR1	NM_053205.1	7– 980	318421	
		7 – 980	318421-C2	
FOS	NM_010234.2	407 – 1427	316921-C3	
GAD1	NM_0080774	62 – 3113	400951-C2	
		62 – 3113	400951-C3	
GAD2	NM_008078.2	552 – 1506	439371-C3	
EGFP	U55763.1	628 – 1352	400281-C3	
SERT	NM_010484.2	452 – 1378	315851-C2	

171

Supplementary Table 2. Detailed statistical imformation

Figure	Sample Size	Statistical test	Statistics
Fig. 1d	n = 12 brain slices from 3 mice	% of VGLUT2-expressing AADC (+) cells	90.6999 %
Fig. 1g	n = 8, 11 mice including 16, 22 brain slices	Two-tailed unpaired *t*-test	t = -9.694<br>df = 13.570<br>*p* = 0.000
Fig. 1h	n = 8, 11 mice including 16, 22 brain slices	Two-tailed unpaired *t*-test	t = -10.226<br>df = 17<br>*p* = 0.000
Fig. 1i	n = 8, 11 mice including 16, 22 brain slices	Two-tailed unpaired *t*-test	t = -9.936<br>df = 11.731<br>*p* = 0.000
Fig. 1k (L)	n = 8, 11 mice including 16, 22 brain slices	Two-tailed unpaired *t*-test	t = -3.274<br>df = 17<br>*p* = 0.004
Fig. 1k (R)	n = 8, 11 mice including 16, 22 brain slices	Two-tailed unpaired *t*-test	t = -3.178<br>df = 17<br>*p* = 0.006
Fig. 2b	n = 14, 15 mice	Two-tailed unpaired *t*-test	t = 3.366<br>df = 27<br>*p* = 0.002
Fig. 2d	n = 5, 6 mice including 27, 30 brain slices	Two-tailed unpaired *t*-test	t = -7.821<br>df = 9<br>*p* = 0.000
Fig. 2f	n = 5, 6 mice including 8, 12 brain slices	Two-tailed unpaired *t*-test	t = -5.466<br>df = 9<br>*p* = 0.000
Fig. 2h	n = 4, 5 mice including 11, 14 brain slices	Two-tailed unpaired *t*-test	t = 7.842<br>df = 7<br>*p* = 0.000
Fig. 2j	n = 7, 5 mice	Two-tailed Mann-Whitney U-test (Base_60 min)	U = 15.000<br>*p* = 0.755
		Two-tailed Mann-Whitney U-test (Base_40 min)	U = 10.000<br>*p* = 0.268
		Two-tailed Mann-Whitney U-test (Base_20 min)	U = 10.000<br>*p* = 0.268
		Two-tailed Mann-Whitney U-test (CNO_20 min)	U = 0.000<br>*p* = 0.003
		Two-tailed Mann-Whitney U-test (CNO_40 min)	U = 5.000<br>*p* = 0.048
		Two-tailed Mann-Whitney U-test (CNO_60 min)	U = 14.000<br>*p* = 0.639
		Two-tailed Mann-Whitney U-test (CNO_80 min)	U = 6.000<br>*p* = 0.073
		Two-tailed Mann-Whitney U-test (CNO_100 min)	U = 3.000<br>*p* = 0.042
		Two-tailed Mann-Whitney U-test (CNO_120 min)	U = 6.000<br>*p* = 0.164
Fig. 3a	n = 4, 4 rats	Two-tailed Mann-Whitney U-test	U = 0.000<br>*p* = 0.029
Fig. 3b	n = 11, 11 mice	Two-tailed unpaired *t*-test	t = 4.032<br>df = 20

			p = 0.001
Fig. 3e	n = 5, 6 mice including 39, 35 brain slices	Two-tailed unpaired t-test	t = 12.091<br>df = 9<br>p = 0.000
Fig. 3g	n = 11, 11 mice	Two-tailed unpaired t-test (0 week)	t = -0.179<br>df = 20<br>p = 0.860
		Two-tailed unpaired t-test (1st week)	t = 0.082<br>df = 20<br>p = 0.936
		Two-tailed unpaired t-test (2nd week)	t = 1.821<br>df = 20<br>p = 0.084
		Two-tailed unpaired t-test (3rd week)	t = 3.212<br>df = 20<br>p = 0.004
		Two-tailed unpaired t-test (4th week)	t = 3.299<br>df = 20<br>p = 0.004
Fig. 3h	n = 18, 18 mice	Two-tailed unpaired t-test	t = -4.556<br>df = 34<br>p = 0.000
Fig. 3i	n = 16, 16 mice	Two-tailed unpaired t-test	t = 5.067<br>df = 18.904<br>p = 0.000
Fig. 3j	n = 16, 16 mice	Two-tailed unpaired t-test	t = 0.920<br>df = 30<br>p = 0.365
Fig. 3l	n = 7, 7 mice including 14, 14 brain slices	Two-tailed unpaired t-test	t = 6.181<br>df = 12<br>p = 0.000
Fig. 3n	n = 7, 6 mice including 15, 14 brain slices	Two-tailed unpaired t-test	t = -5.421<br>df = 11<br>p = 0.000
Fig. 4e	n = 9, 9 cells from 3, 3 mice	Two-tailed unpaired t-test	t = -8.442<br>df = 8.033<br>p = 0.000
Fig. 4g (M)	n = 9, 14 cells from 3, 4 mice	Two-tailed unpaired t-test	t = -2.991<br>df = 17.562<br>p = 0.008
Fig. 4g (R)	n = 10, 14 cells from 3, 4 mice	Two-tailed unpaired t-test	t = -2.613<br>df = 22<br>p = 0.011
Fig. 5b (L)	n = 10 cells from 4 mice	Repeated measure One-way ANOVA followed by contrast test<br>(Between Baseline and Light ON)	F = 79.301<br>p = 0.000
		Repeated measure One-way ANOVA followed by contrast test<br>(Between Light ON and Light OFF)	F = 3.823<br>p = 0.082
Fig. 5b (R)	n = 13 cells from 4 mice	Repeated measure One-way ANOVA followed by contrast test<br>(Between Baseline and Light ON)	F = 0.883<br>p = 0.366
		Repeated measure One-way ANOVA followed by contrast test<br>(Between Light ON and Light OFF)	F = 1.115<br>p = 0.312

Fig. 5d	n = 6 brain slices from 3 mice	% of TAAR1-expressing GAD1 (+) cells	98.4799 %
Fig. 5f(L)	n = 11 cells from 5 mice	Repeated measure One-way ANOVA followed by contrast test (Between Baseline and RO5263397)	F = 217.693<br>*p* = 0.000
		Repeated measure One-way ANOVA followed by contrast test (Between RO5263397 and EPPTB)	F = 10.006<br>*p* = 0.010
Fig. 5f (R)	n = 21 cells from 8 mice	Repeated measure One-way ANOVA followed by contrast test (Between Baseline and RO5263397)	F = 22.443<br>*p* = 0.000
		Repeated measure One-way ANOVA followed by contrast test (Between RO5263397 and EPPTB)	F = 4.944<br>*p* = 0.038
Fig. 5h	n = 7, 10 mice	Two-tailed unpaired *t*-test	t = 3.039<br>df = 15<br>*p* = 0.008
Fig. 6e	n = 34 cells from 2 mice	Repeated measure One-way ANOVA followed by contrast test (Between Baseline and 0-5)	F = 89.934<br>*p* = 0.000
		Repeated measure One-way ANOVA followed by contrast test (Between 0-5 and 10-15)	F = 95.231<br>*p* = 0.000
		Repeated measure One-way ANOVA followed by contrast test (Between Baseline and 10-15)	F = 58.673<br>*p* = 0.000
Fig. 6h	n = 29 cells from 2 mice	Repeated measure One-way ANOVA followed by contrast test (Between Baseline and 0-5)	F = 17.896<br>*p* = 0.000
		Repeated measure One-way ANOVA followed by contrast test (Between 0-5 and 10-15)	F = 27.729<br>*p* = 0.000
		Repeated measure One-way ANOVA followed by contrast test (Between Baseline and 10-15)	F = 0.740<br>*p* = 0.397
Supplementary Fig. 1j (L)	n = 4 mice including 4 brain slices	% of TH-expressing AADC (+) cells in the LHb	1.94 %
Supplementary Fig. 1j (M)	n = 4 mice including 4 brain slices	% of TPH2-expressing AADC (+) cells in the LHb	0.6334 %
Supplementary Fig. 1j (R)	n = 3 mice including 3 brain slices	% of AADC-expressing VGLUT2 (+) cells in the LHb	66.3169 %
Supplementary Fig. 2a	n = 8, 11 mice including 16, 22 brain slices	Two-tailed unpaired *t*-test	t = 0.620<br>df = 8.795<br>*p* = 0.551
Supplementary Fig. 2b	n = 8, 11 mice including 16, 22 brain slices	Two-tailed unpaired *t*-test	t = 1.773<br>df = 17<br>*p* = 0.094
Supplementary Fig. 2c	n = 8, 11 mice including 16, 22 brain slices	Two-tailed unpaired *t*-test	t = -6.007<br>df = 10.138<br>*p* = 0.000

Supplementary Fig. 3b	n = 5, 5, 5, 5 mice including 18, 20, 20, 18 brain slices	One-way ANOVA	F = 13.794<br>$p = 0.000$<br>Tukey HSD<br>Naïve vs TS $p = 0.000$<br>Naïve vs FS $p = 0.010$<br>Naïve vs RS $p = 0.000$
Supplementary Fig. 3c	n = 5, 5, 5, 5 mice including 18, 20, 20, 18 brain slices	One-way ANOVA	F = 15.909<br>$p = 0.000$<br>Tukey HSD<br>Naïve vs TS $p = 0.000$<br>Naïve vs FS $p = 0.007$<br>Naïve vs RS $p = 0.000$
Supplementary Fig. 3e	n = 5, 5, 5, 5 mice including 10, 10, 10, 10 brain slices	One-way ANOVA	F = 14.999<br>$p = 0.000$<br>Tukey HSD<br>Naïve vs TS $p = 0.004$<br>Naïve vs FS $p = 0.000$<br>Naïve vs RS $p = 0.000$
Supplementary Fig. 3f(L)	n = 5, 5, 5, 5 mice including 18, 19, 20, 18 brain slices	One-way ANOVA	F = 1.314<br>$p = 0.304$<br>Tukey HSD<br>Naïve vs TS $p = 0.538$<br>Naïve vs FS $p = 0.287$<br>Naïve vs RS $p = 0.912$
Supplementary Fig. 4c	n = 3 mice including 3 brain slices	% of AADC-expressing Cre-dependent mGFP (+) cells in the LHb	93.8776 %
Supplementary Fig. 4d	n = 7, 7 mice	Two-tailed unpaired *t*-test	t = -1.341<br>df = 12<br>$p = 0.205$
Supplementary Fig. 4e	n = 7, 7 mice	Two-tailed unpaired *t*-test	t = -1.341<br>df = 12<br>$p = 0.205$
Supplementary Fig. 6d	n = 6, 6 mice including 6, 6 brain slices	Two-tailed unpaired *t*-test	t = -15.559<br>df = 5.243<br>$p = 0.000$
Supplementary Fig. 7d	n = 3, 3 HEK293T cell lysates	Two-tailed unpaired *t*-test	t = 3.723<br>df = 4<br>$p = 0.020$
Supplementary Fig. 7f	n = 5, 6 mice including 5, 6 brain slices	Two-tailed unpaired *t*-test	t = 12.191<br>df = 9<br>$p = 0.000$
Supplementary Fig. 8a	n = 11, 11 mice	Two-tailed unpaired *t*-test	t = 1.799<br>df = 20<br>$p = 0.087$
Supplementary Fig. 8b	n = 11, 11 mice	Two-tailed unpaired *t*-test	t = 2.064<br>df = 20<br>$p = 0.052$
Supplementary Fig. 8c	n = 11, 11 mice	Two-tailed unpaired *t*-test	t = 0.400<br>df = 20<br>$p = 0.693$
Supplementary Fig. 8d	n = 11, 11 mice	Two-tailed unpaired *t*-test	t = 0.905<br>df = 20<br>$p = 0.376$

Supplementary Fig. 8e	n = 11, 11 mice	Two-tailed unpaired *t*-test	t = 0.348<br>df = 20<br>*p* = 0.731
Supplementary Fig. 8f	n = 11, 11 mice	Two-tailed unpaired *t*-test	t = -0.219<br>df = 14.983<br>*p* = 0.830
Supplementary Fig. 10e	n = 14, 14 mice	Two-tailed unpaired *t*-test	t = 4.909<br>df = 20.465<br>*p* = 0.000
Supplementary Fig. 10f	n = 14, 14 mice	Two-tailed unpaired *t*-test	t = -2.090<br>df = 26<br>*p* = 0.047
Supplementary Fig. 10g	n = 14, 14 mice	Two-tailed unpaired *t*-test	t = -0.411<br>df = 26<br>*p* = 0.684
Supplementary Fig. 10h	n = 14, 14 mice	Two-tailed unpaired *t*-test	t = -0.108<br>df = 26<br>*p* = 0.915
Supplementary Fig. 10i	n = 14, 14 mice	Two-tailed unpaired *t*-test	t = -0.829<br>df = 26<br>*p* = 0.415
Supplementary Fig. 10j	n = 14, 14 mice	Two-tailed unpaired *t*-test	t = -1.881<br>df = 26<br>*p* = 0.071
Supplementary Fig. 10k	n = 14, 14 mice	Two-tailed unpaired *t*-test	t = -1.255<br>df = 26<br>*p* = 0.221
Supplementary Fig. 10l	n = 14, 14 mice	Two-tailed unpaired *t*-test	t = -1.268<br>df = 26<br>*p* = 0.216
Supplementary Fig. 10m	n = 14, 14 mice	Two-tailed unpaired *t*-test	t = -1.149<br>df = 26<br>*p* = 0.261
Supplementary Fig. 11c	n = 8, 8 mice including 8, 8 brain slices	Two-tailed unpaired *t*-test	t = 6.243<br>df = 8.795<br>*p* = 0.000
Supplementary Fig. 11d	n = 8, 8 mice including 8, 8 brain slices	Two-tailed unpaired *t*-test	t = -4.536<br>df = 8.678<br>*p* = 0.002
Supplementary Fig. 12b	n = 10 cells from 4 mice	Repeated measure One-way ANOVA followed by contrast test<br>(Between Baseline and Light ON)	F = 44.054<br>*p* = 0.000
		Repeated measure One-way ANOVA followed by contrast test<br>(Between Light ON and Light OFF)	F = 5.924<br>*p* = 0.038
Supplementary Fig. 12c	n = 13 cells from 4 mice	Repeated measure One-way ANOVA followed by contrast test<br>(Between Baseline and Light ON)	F = 0.035<br>*p* = 0.855
		Repeated measure One-way ANOVA followed by contrast test<br>(Between Light ON and Light OFF)	F = 1.582<br>*p* = 0.232
Supplementary Fig. 12e	n = 11 cells from 5 mice	Repeated measure One-way ANOVA followed by contrast test<br>(Between Baseline and RO5263397)	F = 25.864<br>*p* = 0.000

		Repeated measure One-way ANOVA followed by contrast test (Between RO5263397 and EPPTB)	F = 23.844<br>p = 0.000
Supplementary Fig. 12f	n = 21 cells from 8 mice	Repeated measure One-way ANOVA followed by contrast test (Between Baseline and RO5263397)	F = 11.887<br>p = 0.003
		Repeated measure One-way ANOVA followed by contrast test (Between RO5263397 and EPPTB)	F = 7.397<br>p = 0.013
Supplementary Fig. 13e (T)	n = 4 mice including 4 brain slices	% of GAD2-expressing GAD1 (+) cells in the RMTg	97.0213 %
Supplementary Fig. 13e (M)	n = 3 mice including 3 brain slices	% of VGLUT2-expressing GAD1 (+) cells in the RMTg	0.8984 %
Supplementary Fig. 13e (B)	n = 3 mice including 3 brain slices	% of VGLUT3-expressing GAD1 (+) cells in the RMTg	0.3057 %
Supplementary Fig. 13g	n = 3 mice including 3 brain slices	% of TAAR1-expressing AADC (+) cells in the LHb	0.1608 %
Supplementary Fig. 14b	n = 5, 5 mice including 8, 10 brain slices	Two-tailed unpaired *t*-test	t = -2.183<br>df = 8<br>p = 0.061
Supplementary Fig. 14d	n = 5, 5, 5, 5 mice including 9, 7, 9, 10 brain slices	One-way ANOVA	F = 0.218<br>p = 0.883<br>Tukey HSD<br>Naïve vs TS p = 0.992<br>Naïve vs FS p = 1.000<br>Naïve vs RS p = 0.958
Supplementary Fig. 14f	n = 5, 5 mice including 8, 8 brain slices	Two-tailed unpaired *t*-test	t = 0.385<br>df = 8<br>p = 0.710
Supplementary Fig. 15d	n = 10 cells from 6 mice	Repeated measure One-way ANOVA followed by contrast test (Between Baseline and Light-ON)	F = 22.864<br>p = 0.000
		Repeated measure One-way ANOVA followed by contrast test (Between Light-ON and Light-ON+EPPTB)	F = 25.705<br>p = 0.000
Supplementary Fig. 15e	n = 10 cells from 6 mice	Repeated measure One-way ANOVA followed by contrast test (Between Baseline and Light-ON)	F = 11.795<br>p = 0.007
		Repeated measure One-way ANOVA followed by contrast test (Between Light-ON and Light-ON+EPPTB)	F = 20.214<br>p = 0.001
Supplementary Fig. 15h	n = 14 cells from 7 mice	Repeated measure One-way ANOVA followed by contrast test (Between Baseline and EPPTB)	F = 10.518<br>p = 0.006
		Repeated measure One-way ANOVA followed by contrast test (Between EPPTB and EPPTB+Light-ON)	F = 3.177<br>p = 0.098
Supplementary Fig. 15i	n = 14 cells from 7 mice	Repeated measure One-way ANOVA followed by contrast test (Between Baseline and EPPTB)	F = 7.702<br>p = 0.016

		Repeated measure One-way ANOVA followed by contrast test<br>(Between EPPTB and EPPTB+Light-ON)	F = 0.526<br>*p* = 0.481
Supplementary Fig. 16b	n = 12, 20 mice	Two-tailed unpaired *t*-test	t = -2.368<br>df = 17.066<br>*p* = 0.030
Supplementary Fig. 17b	n =34, 103 cells from 2 mice	Two-tailed unpaired *t*-test	t = 18.678<br>df = 99.322<br>*p* = 0.000
Supplementary Fig. 17e	n = 29, 53 cells from 2 mice	Two-tailed unpaired *t*-test	t = 16.290<br>df = 78.307<br>*p* = 0.000
Supplementary Fig. 17g	n = 34, 29 cells from 2, 2 mice	Two-tailed unpaired *t*-test	t = -4.863<br>df = 43.389<br>*p* = 0.000

(L), left panel; (R), right panel; (M) middle panel; (T), top panel; (B), bottom panel
