# Supplementary material for: Neural mechanism of acute stress regulation by trace aminergic signalling in the lateral habenula in male mice
Source: Nat Commun. 2023 Apr 27;14:2435. doi: 10.1038/s41467-023-38180-7 (PMC10140019; doi:10.1038/s41467-023-38180-7)
Supplement: Supplementary file 3 — Description of Additional Supplementary Files [file 41467_2023_38180_MOESM3_ESM.pdf]

Description of Additional Supplementary Files

File Name: Supplementary Movie 1
Description: Anterograde tracing of habenula D-neurons
