# Supplementary material for: Neural mechanism of acute stress regulation by trace aminergic signalling in the lateral habenula in male mice
Source: Nat Commun. 2023 Apr 27;14:2435. doi: 10.1038/s41467-023-38180-7 (PMC10140019; doi:10.1038/s41467-023-38180-7)
Supplement: Supplementary file 5 — Reporting Summary [file 41467_2023_38180_MOESM5_ESM.pdf]

nature portfolio

Corresponding author(s): Hyun Kim

Last updated by author(s): Apr 6, 2023

Reporting Summary

Nature Portfolio wishes to improve the reproducibility of the work that we publish. This form provides structure for consistency and transparency in reporting. For further information on Nature Portfolio policies, see our Editorial Policies and the Editorial Policy Checklist.

Statistics

For all statistical analyses, confirm that the following items are present in the figure legend, table legend, main text, or Methods section.

n/a	Confirmed	
☐	☒	The exact sample size (n) for each experimental group/condition, given as a discrete number and unit of measurement
☐	☒	A statement on whether measurements were taken from distinct samples or whether the same sample was measured repeatedly
☐	☒	The statistical test(s) used AND whether they are one- or two-sided<br>*Only common tests should be described solely by name; describe more complex techniques in the Methods section.*
☒	☐	A description of all covariates tested
☐	☒	A description of any assumptions or corrections, such as tests of normality and adjustment for multiple comparisons
☐	☒	A full description of the statistical parameters including central tendency (e.g. means) or other basic estimates (e.g. regression coefficient) AND variation (e.g. standard deviation) or associated estimates of uncertainty (e.g. confidence intervals)
☐	☒	For null hypothesis testing, the test statistic (e.g. F, t, r) with confidence intervals, effect sizes, degrees of freedom and P value noted<br>*Give P values as exact values whenever suitable.*
☒	☐	For Bayesian analysis, information on the choice of priors and Markov chain Monte Carlo settings
☒	☐	For hierarchical and complex designs, identification of the appropriate level for tests and full reporting of outcomes
☐	☒	Estimates of effect sizes (e.g. Cohen's d, Pearson's r), indicating how they were calculated

Our web collection on statistics for biologists contains articles on many of the points above.

Software and code

Policy information about availability of computer code

Data collection	TCS SP8 Dichroic/CS microscope (Leica)<br>Cryotome (Leica CM300, Leica)<br>Analyst software version 1.7 (SCIEX)<br>EthoVision XT 12.0.1136 tracking software (Noldus)<br>ExionLC™ / QTRAP® 6500+ LC-MS/MS System (SCIEX)<br>z.1 light sheet fluorescence microscope (Carl Zeiss)<br>Digital Lynx recording system (Neuralynx)<br>MultiClamp 700B amplifier (Molecular Devices)<br>Digidata 1440 digitiser(Molecular Devices)<br>pClamp 10 software (Molecular Devices)
Data analysis	EthoVision XT 12.0.1136 tracking software (Noldus)<br>HALO v2.3.2089.18 (Indica Labs)<br>Imaris software (Bitplane)<br>Clampfit 10 (Molecular Devices)<br>ExionLC™ / QTRAP® 6500+ LC-MS/MS System (SCIEX)<br>Matlab software R2017a (MathWorks)<br>Offline Sorter (Plexon Inc.)<br>SPSS Statistics 26 for Windows (IBM)

For manuscripts utilizing custom algorithms or software that are central to the research but not yet described in published literature, software must be made available to editors and reviewers. We strongly encourage code deposition in a community repository (e.g. GitHub). See the Nature Portfolio guidelines for submitting code & software for further information.

Data

Policy information about availability of data

All manuscripts must include a data availability statement. This statement should provide the following information, where applicable:

- Accession codes, unique identifiers, or web links for publicly available datasets
- A description of any restrictions on data availability
- For clinical datasets or third party data, please ensure that the statement adheres to our policy

All source data are provided with the manuscript. Additional data are available from the corresponding author upon reasonable request. Databases used for this study include the Allen Brain Atlas (Ddc, https://mouse.brain-map.org/experiment/show/79556614).

Human research participants

Policy information about studies involving human research participants and Sex and Gender in Research.

Reporting on sex and gender	Not applicable
Population characteristics	Not applicable
Recruitment	Not applicable
Ethics oversight	Not applicable

Note that full information on the approval of the study protocol must also be provided in the manuscript.

Field-specific reporting

Please select the one below that is the best fit for your research. If you are not sure, read the appropriate sections before making your selection.

☒ Life sciences ☐ Behavioural & social sciences ☐ Ecological, evolutionary & environmental sciences

For a reference copy of the document with all sections, see nature.com/documents/nr-reporting-summary-flat.pdf

Life sciences study design

All studies must disclose on these points even when the disclosure is negative.

Sample size	No statistical methods to pre-determine sample size were used. Sample sizes were based on previous studies in the field of behavioural neuroscience (Han et al 2017 Sci Rep doi.org/10.1038/s41598-017-01088-6, Yoo et al 2021 Sci Rep doi.org/10.1038/s41598-021-83310-0)
Data exclusions	Viral expression of transgenes and implant placement were verified by histology before the data were included in the analysis.
Replication	In vivo and in vitro experiments were conducted independently at least two times to ensure reproducibility. All experiments were repeated as indicated in the text and/or figure legends to obtain consistent and reliable findings.
Randomization	Allocation of animals were random.
Blinding	In all behavioural experiments, the experimenter was blinded to the animals' genotypes and the experimental conditions; the data were analysed in a blinded manner as well.<br>In imaging and eletrophysiology experiments, data collection was not blinded to experimenters but data analysis was blinded to experimenters.

Reporting for specific materials, systems and methods

We require information from authors about some types of materials, experimental systems and methods used in many studies. Here, indicate whether each material, system or method listed is relevant to your study. If you are not sure if a list item applies to your research, read the appropriate section before selecting a response.

Materials & experimental systems

n/a	Involved in the study
☐	☒ Antibodies
☐	☒ Eukaryotic cell lines
☒	☐ Palaeontology and archaeology
☐	☒ Animals and other organisms
☒	☐ Clinical data
☒	☐ Dual use research of concern

Methods

n/a	Involved in the study
☒	☐ ChIP-seq
☒	☐ Flow cytometry
☒	☐ MRI-based neuroimaging

Antibodies

Antibodies used

1. anti c-Fos (1:500; #SC-52G, Santa Cruz Biotechnology, Inc.)
2. anti-GABA (1:500; #A2052, Sigma-Aldrich)
3. Alexa Fluor® 488 donkey anti-goat (1:500; #A11055, Thermo Fisher Scientific)
4. Alexa Fluor® 647 donkey anti-rabbit (1:500; #715- 605-152, Jackson Immuno Research Inc.)
5. Hoechst (1:1000; #H3570, Thermo Fisher Scientific)
6. anti-GFP (1:1000; sc-9996, Santa Cruz Biotechnology, Inc.)
7. anti-DOPA decarboxylase (1:1000; ab3905, Abcam)
8. anti-GFP (1:5000; ab290, Abcam)
9. beta-actin (1:5000; sc-47778, Santa Cruz Biotechnology, Inc.)

Validation

1. https://www.scbt.com/p/c-fos-antibody-4
2. https://www.sigmaaldrich.com/KR/ko/product/sigma/a2052?gclid=EAIaIQobChMI3MyQp_Xa_QIVl56WCh0ecw_0EAAYASAAEgKmaPD_BwE&gclsrc=aw.ds
3. https://www.thermofisher.com/antibody/product/Donkey-anti-Goat-IgG-H-L-Cross-Adsorbed-Secondary-Antibody-Polyclonal/A-11055
4. https://www.jacksonimmuno.com/catalog/products/711-605-152
5. https://www.thermofisher.com/order/catalog/product/kr/en/H3570
6. https://www.scbt.com/p/gfp-antibody-b-2
7. https://www.abcam.com/products/primary-antibodies/dopa-decarboxylaseddc-antibody-ab3905.html
8. https://www.abcam.com/products/primary-antibodies/gfp-antibody-ab290.html
9. https://www.scbt.com/p/beta-actin-antibody-c4

Eukaryotic cell lines

Policy information about cell lines and Sex and Gender in Research

Cell line source(s): HEK293T cells were used for production of recombinant AAV vectors.

Authentication: The cell line was not authenticated.

Mycoplasma contamination: The cell line was not tested for mycoplasma contamination.

Commonly misidentified lines (See ICLAC register): No commonly misidentified lines were used in the study.

Animals and other research organisms

Policy information about studies involving animals; ARRIVE guidelines recommended for reporting animal research, and Sex and Gender in Research

Laboratory animals: Adult male C57BL/6J mice and Sprague–Dawley rats (7 weeks of age) were purchased from Japan SLC Inc. (Shizuoka). AADCCre (B6.FVB(Cg)-Tg(Ddc-cre)SD56Gsat/Mmucd, RRID:MMRRC_037410-UCD) mice were obtained from the Mutant Mouse Resource and Research Center (MMRRC) of the University of California-Davis (Davis). VGATCre (B6J.129S6(FVB)-Slc32a1tm2(cre)Lowl/MwarJ, RRID:IMSR_JAX:028862) mice and with Ai14 (B6. Cg-Gt(ROSA)26Sortml4(CAG-tdTomato)Hze/J, RRID:IMSR_JAX:007914) mice were obtained from the Jackson Laboratory. All mice used for experiments were heterozygotes maintained on the C57BL/6J background. All animals were housed three to four per cage under a 12/12-h light/dark cycle (lights on at 8 a.m.), with a room temperature of 22° C and humidity of 50%.

Wild animals: No wild animals were used.

Reporting on sex: We excluded female mice from the experiments because they have the estrous cycle that affects behaviours such as stress response, anxiety and depression.
Luine V, Frankfurt M. (2013) Interactions between estradiol, BDNF and dendritic spines in promoting memory. Neuroscience. 239:34-45.
Kokras N, Antoniou K, Mikail HG, Kafetzopoulos V, Papadopoulou-Daifoti Z, Dalla C. (2015) Forced swim test: What about females? Neuropharmacology. 99:408-21.

Field-collected samples	No field collected samples were used in the study.
Ethics oversight	All experimental procedures were performed in compliance with the Guide for the Care and Use of Laboratory Animals from the Korea University, and approved by the Korea University Institutional Animal Care and Use Committee.

Note that full information on the approval of the study protocol must also be provided in the manuscript.
